# A Case of Multiple Myeloma Coexisting with Primary Hyperparathyroidism and Review of the Literature

**DOI:** 10.1155/2013/420565

**Published:** 2013-02-26

**Authors:** Nasir Hussain, Moona Khan, Aparna Natarajan, Mubeenkhan Mohammedabdul, Usman Mustafa, Kalpana Yedulla, Aibek E. Mirrakhimov

**Affiliations:** Saint Joseph Hospital, Resurrection Health Care, Department of Internal Medicine, 2900 North Lake Shore Drive, Chicago, IL 60657, USA

## Abstract

Hypercalcemia is a common medical problem with an estimated prevalence of 15% among hospitalized patients. Multiple myeloma (MM) and primary hyperparathyroidism (PHPT) are among the most common causes of hypercalcemia but coexistence of both pathologic processes in a patient is an extremely rare phenomenon. In this paper we have discussed a patient presenting with this rare phenomenon. We have also provided a comprehensive review of the scientific literature published on codiagnosis of MM and PHPT.

## 1. Introduction

Hypercalcemia is a common clinical problem with an estimated prevalence of 15% among hospitalized patients [31]. The etiology of hypercalcemia is complex with many factors playing a pathogenic role. From a clinical standpoint, it may present with changes in mental status, generalized weakness, polyuria, and constipation. Multiple myeloma (MM) and primary hyperparathyroidism (PHPT) are among the most common causes of hypercalcemia but coexistence of the two pathologic processes in one patient is an extremely rare phenomenon. In this paper, we have discussed a patient presenting with this rare phenomenon and have reviewed the relevant scientific literature. 

## 2. Case Presentation

A 92-year-old Caucasian female with a past medical history of Alzheimer's dementia, seizure disorder, osteoporosis, and osteoarthritis was admitted to the hospital for an evaluation of a new onset confusion and constipation. Review of symptoms during admission was significant for anorexia, weight loss, constipation for the last three weeks, and history of a fall one month prior to the presentation. Family history was significant for MM in a sister. At the time of presentation, the patient was using donepezil, memantine, vitamin D with calcium, calcium carbonate (calcium containing antacid), and levetiracetam. Vitals at the time of admission were blood pressure 140/58, pulse 68, respiratory rate 18, oxygen saturation 98% on room air, and temperature 97.4. On physical examination, the patient was alert and oriented in place and person but not in time. Other significant findings were diastolic murmur in right second intercostal space, petechiae over lower extremities, and back tenderness, which the patient attributed to a recent fall. Lumbar spine X-ray was done three weeks prior to the presentation that showed degenerative changes with no evidence of fracture. Basic blood workup including complete blood count and comprehensive metabolic panel was done, which revealed anemia, leucopenia, and hypercalcemia. Home medications were held for concerns of hypercalcemia and confusion. 

**Figure 1:**
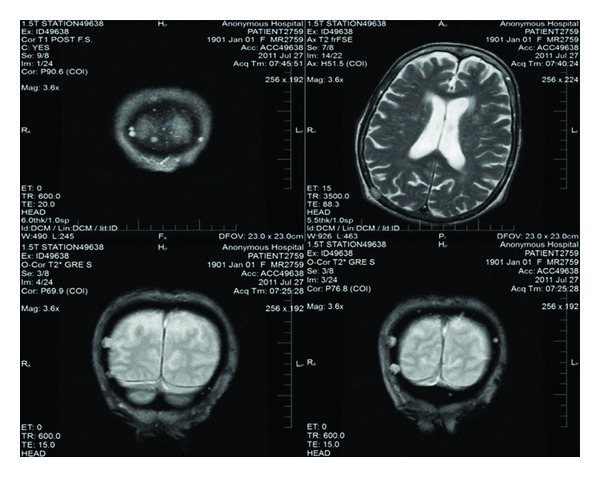
MRI demonstrating lytic lesions.

**Figure 2:**
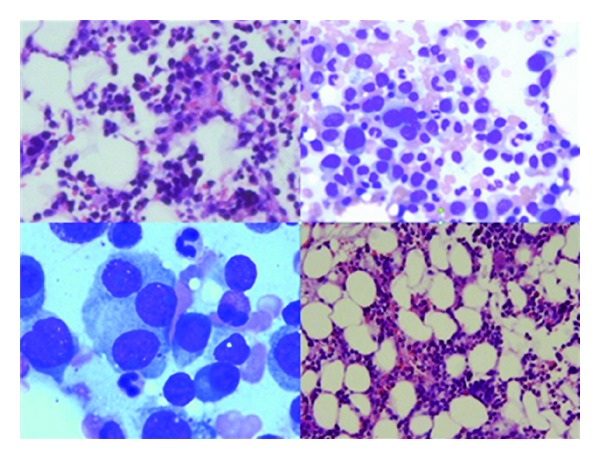
Mildly hypercellular bone marrow with plasmacytosis (30%), consistent with multiple myeloma.

**Figure 3:**
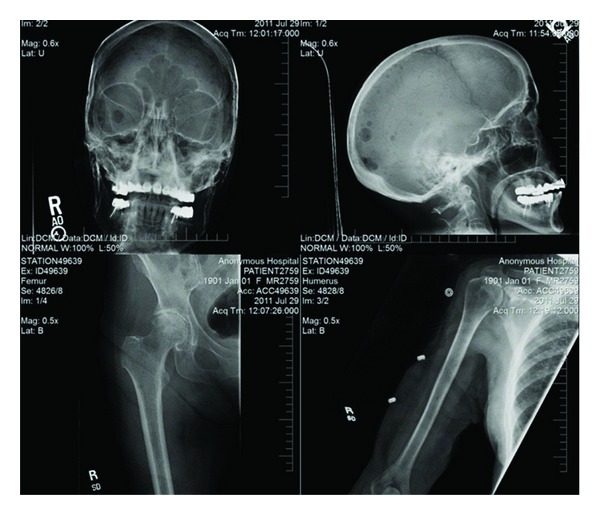
Skeletal survey showing lytic lesions in long bones and skulls.

Endocrinology and neurology services were consulted. MRI of the brain was done, which showed lytic lesions as shown in Figure 1. MM was suspected; serum protein electrophoresis (SPEP), urine protein electrophoresis (UPAP) and bone marrow biopsy were done which confirmed the diagnosis of MM (IgG kappa) (International Staging System stage II). Bone marrow biopsy showed mildly hypercellular bone marrow with plasmacytosis (30%) as shown in Figure 2. Skeletal survey showed diffuse lytic lesions throughout long bones, pelvis, and skull (Figure 3). Surprisingly, intact PTH came back high suggesting primary hyperparathyroidism (PHPT). The data on laboratory tests are presented in Table 1.

Hypercalcemia was managed with intravenous hydration, calcitonin, bisphosphonates, and furosemide. The patient was started on melphalan and prednisone, which were later switched to lenalidomide with a high dose of dexamethasone due to a poor treatment response. After one and a half year, the patient is still following in our outpatient oncology center being on a low dose of lenalidomide with a stable M protein. 

## 3. Discussion

Hypercalcemia is common in patients with MM and occurs in 28% of myeloma cases [32]. MM may cause hypercalcemia through multiple mechanisms. First, plasma cells produce various cytokines, including TNF-*β* and IL-6, that activate osteoclasts and lead to calcium washout from bones to the bloodstream [33]. Second, some studies suggest that MM cells may secrete parathyroid hormone-related peptide similarly to other malignancies, such as squamous cell lung carcinoma [34, 35]. Third, serum calcium may be falsely elevated because of a binding to immunoglobulin [36, 37].

Clubb et al. [38] described first-case linking PHPT and paraproteinemia in 1964. Drezner and Lebovitz were the first who described a case of concomitant MM and PHPT in 1979 [30]. Some researchers speculate that the association between MM and PHTP may not be coincidental [39, 40], although mechanisms explaining codiagnosis are not known. Arnulf et al. showed that the prevalence of monoclonal gammopathy is higher in patients with PHTP as compared to general population [40]. Pest et al. hypothesized that elevated PTH may mediate the induction of MM through the downstream biological effects of IL-6 [1]. This hypothesis was supported by the study performed by Pirih et al., who showed that PTH decreases apoptotic cell death of the hematopoietic stem cells via the IL-6 [41].

PHPT leads to hypercalcemia via direct bone resorption [42] mediated by osteoclasts. Another important mechanism is through an increased calcium absorption in the duodenum and greater reabsorption in the kidneys.

The above-mentioned pathogenic mechanism gives an insight to how PHPT and MM may be linked. Some studies have suggested that calcium may act as a mitogenic factor [43], whereas others suggest that myelomatous proteins may interfere with polypeptide hormone synthesis bind their circulating fractions, and/or block their peripheral effects that may secondarily stimulate parathyroid gland [29]. However, both of these diseases are common among elderly and may share similar risk factors, such as ionizing radiation [44, 45], and a simple coincidence may be the case. 

Summary of published cases [1–28] is presented in Table 2. Codiagnosis of PHPT and MM should be suspected in cases of difficult-to-control hypercalcemia. Most of the cases of coexistent MM and PHPT have been observed in females (23 out of 29 reported cases). The youngest patient with codiagnosis was a 45-year-old female and the oldest patient was a 92-year-old female. PHPT is more common in females, whereas the opposite is true for MM. Differences in incidence of the two diseases may explain female preponderance (MM less frequent than PHPT). Initial diagnosis was highly variable, eleven cases had primary diagnosis of hyperparathyroidism, ten had primary diagnosis of MM and seven had both diagnosis made at presentation. The type of immunoglobulin chains of MM observed in all the cases was variable as six patients had light chain MM, remaining patients had a combination of heavy and light chain MM, one patient had nonsecretory type of MM. All the patients had calcium ≥11 mg/dL at the time of presentation. Majority of patients had parathyroid adenoma as a cause of PHPT, few had chief cell hyperplasia, and none had parathyroid cancer. Parathyroidectomy, combination of radiotherapy, and chemotherapy had been used for treatment of this coexistent condition with variable success. Rao et al. [2] suggested that parathyroidectomy in patients with coexistent PHPT and MM serves three folds; first, it removes confusion about etiology of hypercalcemia; second, it alters prognosis of myeloma; third, calcium can be used as a tumor marker in cases if there is a recurrence of tumor. Considering age, our patient was not a candidate for surgery, in such patient population medical alternative to parathyroidectomy is needed. Ten out of 29-patients died within 5 years after codiagnosis, and out of those ten, eight died within one year. 

## 4. Conclusions

A search for concomitant cause of hypercalcemia should be pursued in cases of difficult-to-control hypercalcemia and in elderly individuals, in whom the incidence of PTHP and MM is common. 

## Figures and Tables

**Table 1:** 

Result name	Results	Reference range
WBC	2.9 K/mm cu	4.2–11.0
Platelet	156 K/mm cu	140–400
Hemoglobin Hb	9.0 g/dL	12.0–15.0
Hematocrit	27.1%	36.0–47.0
Reticulocyte	0.7%	0.5–2.8
Blood urea nitrogen	22 mg/dL	5–20
Creatinine	1.11 mg/dL	0.0–1.00
Sodium	143 mmol/L	135–145
Potassium	4.0 mmol/L	3.4–5.1
Chloride	104 mmol/L	98–109
Bicarbonate	33 mmol/L	23–31
Calcium	13.3 mg/dL	8.4–10.5
Total protein	7.0 g/dL	6.4–8.3
Albumin	4.0 g/dL	3.4–5.2
Aspartate amino transferase	20 IU/L	0–32
Alanine amino transferase	10 IU/L	0–40
Alkaline phosphatase	67 IU/L	35–104
Bilirubin total	0.2 mg/dL	0–10.0
Haptoglobin	157 mg/dL	36–195
Vitamin B12	532 pg/mL	211–946
TSH	1.160 uIU/mL	0.400–5.400
Vitamin D25 OH	47.0 ng/mL	30.0–100.0
25 Hydroxy D3	26 pg/mL	
25 hydroxy D2	<8	
Vitamin D 1,25(OH)2	26	18–72
Folate	>20.0 ng/mL	3.1–17.5
Ferritin	64 ng/mL	13–150
Phosphorous	2.8 mg/dL	2.0–4.0
Lactate dehydrogenase	137 IU/L	135–214
Total iron	30 ug/dL	30–160
Unsaturated IBC	234.0 ug/dL	110.0–370.0
Total IBC	264.0	228.0–428.0
Percentage of iron saturation	11%	20–55
PTH intact on day of presentation	70.5 pg/mL	15.0–65.0
PTH 7 months later	540.0 pg/mL	
PTH-related protein	18 pg/mL	14–27
Beta-2 microglobulin	3.3 mg/L (5.8 mg/L four months later)	0.8–2.2
Serum viscosity	1.5 relative to H_2_O	1.5–1.9
PT/INR	10.6/1.0 sec	9.211.8/0.9–1.1
APTT	29 sec	24–33
Immunoglobulins		
IGA	29 mg/dL	50–400
IGG	692 mg/dL (1200, 5 months later)	600–1500
IGM	6 mg/dL	50–300
Free kappa light chains	1510	3.3–19.4 mg/L
Free lambda light chains	2.4	5.7–26.3
Free Kappa/lambda	629.17	0.26–1.65
Urine protein electrophoresis		
Urine volume 24 hours	1150 mL/24 hour	
Urine-protein electrophoresis (UPE)	253 mg/24 hour	0–165
Albumin UPE	30.6%	
Alpha1	16.1%	
Alpha2	14.1%	
Beta	17.1%	
Gamma	22.1%	
Immunofixation	Free kappa light chains	
24-hour-urine protein	310.5 mg/24 hour	0–150
24-hours-urine creatinine	0.7 g/24 hour	0.74–1.57
24-hour-urine volume	1150 cc	
24-hour-urine creatinine	0.5 g/24 hour	0.74–1.57
24-hour-urine volume	900 mL (repeat test)	
24-hour-urine calcium	239 mg/24 hour	100–300
Serum protein electrophoresis		
Albumin	3.3 g/dL	3.1–5.0
Alpha 1	0.3 g/dL	0.2–0.5
Alpha2	0.7 g/dL	0.5–1.1
Beta	0.6 g/dL	0.6–1.1
Gamma	1.5 g/dL	0.7–1.7
Albumin/globulin	1.0	
M spike	1.09 g/dL	
Total protein	6.5 g/dL	6.4–8.3
Immunofixation	Monoclonal paraprotein of class IgG kappa	
CD56 NK cells	63%	3–35
CD 138 marker	26%	
Lambda B-cell marker	1%	1–7
Kappa B-cell marker	73%	2–14%
CD45 LCA	98%	92–100
CD38 Marker	26%	1–17

*Pathology.* Normal female bone marrow karyotype. No clonal, structural, or numerical chromosome abnormalities identified. FISH analysis indicates normal hybridization signals with MM probe panel. This excludes majority of chromosome rearrangements known to be associated with MM.

*Surgical Pathology.* Mildly hyper cellular bone marrow with plasmacytosis consistent with MM.

*Leukemia/lymphoma panel*. Bone marrow aspirate shows 30–40% plasma cells with kappa light chain restrictions. (plasma cell dyscrasia).

*Peripheral Smear.* Lymphocytes with foamy cytoplasm, no rouleaux formation, adequate polys with occasional platelet clumps.

**Table 2:** 

*n*	Author	Age/ Gender	Type of MM	Ca (mg/dL)	Therapy for MM and PHPT	Parathyroid histology	Outcome	Initial diagnosis
1	Pest et al. [1]	76 F	IgA-?	13.2	Hydration, bisphosphonates, Lasix, melphalan, cyclophosphamide, and steroids	Adenoma	Survived	PHPT
2	Rao et al. [2]	54 M	IgG-lambda	11.2	Adriamycin, melphalan, prednisone, cyclophosphamide, and parathyroidectomy	Adenoma	Died after 12 years	Both
3	Jackson and Orland [3]	45 F	IgG-lambda	17.1	Hydration, Lasix, prednisone, and melphalan	Adenoma	—	MM
4	Chisholm et al. [4]	80 M	Kappa	13.1	Parathyroidectomy, radiotherapy, melphalan, prednisone, vincristine, carmustine, cyclophosphamide, hydration, and Lasix	Adenoma (c-cells)	Died 2 years later	PHPT
5	Francis et al. [5]	70F	Lambda	11.6	Norethisterone, vincristine, melphalan, and prednisone	Adenoma	Died 3 weeks later	PHPT
6	Mundis and kyle [6]	76 F	IgG-kappa	11.0	Melphalan, prednisone, and parathyroidectomy	Adenoma (c-cells)	survived	MM
7	Stone et al. [7]	47 F	IgA-kappa	13.7	Melphalan, prednisone, radiotherapy, parathyroidectomy, hydration, and mithramycin	Adenoma	Died	MM
8	Hoelzer and Silverberg [8]	51 F	IgA-lambda	11.9	Parathyroidectomy?	Adenoma (c-cells)	—	PHPT
9	Schneider and Thomas [9]	74 F	IgG-kappa	12.0	Melphalan, prednisone, and parathyroidectomy	Adenoma	Survived	MM
10	Toussirot et al. [10]	82 M	Kappa	15.2	Melphalan, prednisone, and parathyroidectomy	Hyperplasia	Died	PHPT
11	Goto et al. [11]	73 F	Kappa	13.2	Parathyroidectomy, melphalan	Adenoma	Died 1 year later	PHPT
12	Otsuka et al. [12]	77 F	IgG-lambda		Melphalan, prednisone, bisphosphonates, calcitonin, and parathyroidectomy	c-cells hyperplasia	Survived	—
13	Fery-Blanco et al. [13]	68 F	IgG-kappa	11.28	? chemotherapy and surgery refused	Adenoma	Died	Both
14	Sarfati et al. [14]	62 F	IgA-kappa	16.4	Mithramycin, lasix, plasmaphoresis, Adriamycin, vincristine, prednisone, and parathyroidectomy	Adenoma	Survived	MM
15	Rosen et al. [15]	81 M	IgG-kappa	13.4	Hydration, bisphosphonates, melphalan, prednisone, radiotherapy, needle aspiration of parathyroid gland, and refused surgery	Adenoma	Survived	MM
16	Tomon et al. [16]	60 F	IGA-kappa	—	—	—	—	MM
17	Fanari et al. [17]	59 F	lambda	12.7	Hydration, bisphosphonates, cinacalcet, bortezomib and dexamethasone	Possible Adenoma	Died 4 months later	Both
18	Bogas et al. [18]	72 F	IgG-kappa	13.66	Melphalan, prednisone, and Interferon?	Adenoma	Died 4 years later	Both
19	Katayama et al. [19]	50 F						PHPT
20	Romagnoli et al. [20]	70 F	—	—	Parathyroidectomy, steroids and chemotherapy	Adenoma	—	PHPT (MEN-1)
21	Toh and Winocour et al. [21]	71 M		12.0	Melphalan, prednisone, and bisphosphonates		Died 6 weeks later	MM
22	Sope$\tilde{\text{n}}$a et al. [22]	77 F	Kappa (ns)	12.9	Bisphosphonates, refused surgery, or chemotherapy		Died 1 year later	Both
23	Khandwala and Boctor [23]	72 F	—	11.7/ 16.6*	Parathyroidectomy, bisphosphonates, calcitonin, melphalan, and prednisone	Adenoma	—	PHPT
24	Patel et al. [24]	73 F	IgG- kappa	13.5	Bisphosphonates, steroids, thalidomide, plicamycin, and parathyroidectomy	Adenoma	—	MM
25	Avcioglu et al. [25]	52 F	IgG-kappa	12.6	Parathyroidectomy and steroids	Adenoma	—	Both
26	Chowdhury and Scarsbrook et al. [26]	87 F	—	—	—	—	—	PHPT
27	Dalgleish and Gatenby [27]	59 F	IgG-lambda	11.68	Hydration, lasix, prednisone, mithramycin, cyclophosphamide, and parathyroidectomy	Adenoma	Survived	MM
28	Peters et al. [28]	73 M	IgA-lambda	16	Parathyroidectomy, chemotherapy, and radiotherapy	Hyperplasia	Died 1 week later	PHPT
29	Our case	92 F	IgG-kappa	13 .3	Bisphosphonates, Lasix, hydration, calcitonin, melphalan, prednisone, lenalidomide, and dexamethasone	—	Survived	Both
30	Johansson and Werner [29] mentioned 3 cases of MM and PHPT (no detail of the cases is given), one other such as has been described by Drezner and Lebovitz [30] without much detail.							

*Calcium at time of diagnosis of MM.

## References

[1] Pest EP, McQuaker G, Hunter JA, Moffat D, Stanley AJ (2005). Primary hyperparathyroidsm, amyloid and multiple myeloma: an unusual association. *Scottish Medical Journal*.

[2] Rao DS, Antonelli R, Kane KR, Kuhn JE, Hetnal C (1991). Primary hyperparathyroidism and monoclonal gammopathy. *Henry Ford Hospital Medical Journal*.

[3] Jackson RM, Orland MJ (1979). Parathyroid adenoma in a patient with multiple myeloma. *Southern Medical Journal*.

[4] Chisholm RC, Weaver YJ, Chung EB, Townsend JL (1981). Parathyroid adenoma and light chain myeloma. *Journal of the National Medical Association*.

[5] Francis RM, Bynoe AG, Gray C (1982). Hypercalcaemia due to the coexistence of parathyroid adenoma and myelomatosis. *Journal of Clinical Pathology*.

[6] Mundis RJ, Kyle RA (1982). Primary hyperparathyroidism and monoclonal gammopathy of undetermined significance. *American Journal of Clinical Pathology*.

[7] Stone MJ, Lieberman ZH, Chakmakjian ZH, Matthews JL (1982). Coexistent multiple myeloma and primary hyperparathyroidism. *Journal of the American Medical Association*.

[8] Hoelzer DR, Silverberg AB (1984). Primary hyperparathyroidism complicated by multiple myeloma. *Archives of Internal Medicine*.

[9] Schneider W, Thomas M (1989). Hypercalcaemia in coexistent parathyroid adenoma and multiple myeloma: problems of differential diagnosis. *Deutsche Medizinische Wochenschrift*.

[10] Toussirot E, Bille F, Henry JF, Acquaviva PC (1994). Coexisting kappa light chain multiple myeloma and primary hyperparathyroidism. *Scandinavian Journal of Rheumatology*.

[11] Goto S, Yoshioka M, Nagai K (1995). Primary hyperparathyroidism associated with multiple myeloma. *Internal Medicine*.

[12] Otsuka F, Hayakawa N, Ogura T (1997). A case of primary hyperparathyroidism accompanying multiple myeloma. *Endocrine Journal*.

[13] Fery-Blanco C, Prati C, Ornetti P (2007). Hypercalcemia ofdouble origin: association ofmultiple myeloma andectopic parathyroidal adenoma. *Revue de Medecine Interne*.

[14] Sarfati E, de Ferron P, Dubost C, Assens P, Veyssier P, Detour B (1985). Multiple myeloma associated with primary hyperparathyroidism caused by an adenoma. *Annales de medecine interne*.

[15] Rosen C, Segal H, Hartz CE, Mroz F, Carlton E (1992). Primary hyperparathyroidism in an elderly patient with multiple myeloma. *Journal of the American Geriatrics Society*.

[16] Tomon M, Fukase M, Nakata M (1989). A case of multiple myeloma associated with primary hyperparathyroidism. *Hormone to Rinsho*.

[17] Fanari Z, Kadikoy H, Haque W, Pacha O, Abdellatif A (2010). Medical management of primary hyperparathyroidism with concommitant multiple myeloma. *Internal Medicine*.

[18] Bogas M, Costa L, Araújo D (2008). Coexistence of primary Hyperparathyroidism and multiple myeloma; association and rare manifestation. *Acta Reumatologica Portuguesa*.

[19] Katayama Y, Matsuda H, Katoh Y (1989). Multiple myeloma in a patient with primary hyperparathyroidism. *Hinyokika Kiyo*.

[20] Romagnoli E, Minisola S, Carnevale V, Spagna G, D’Erasmo E, Mazzuoli G (1990). Coexistent multiple myeloma and MEN type 1. *Postgraduate Medical Journal*.

[21] Toh V, Winocour P (2000). Multiple myeloma with hyperparathyroidism. *Hospital Medicine*.

[22] Sopeña B, Rodríguez GJ, De La Fuente J, Martínez-Vázquez C (2004). Two causes of hypercalcemia: learning by the holmesian method. *Mayo Clinic Proceedings*.

[23] Khandwala HM, Boctor MA (2004). Multiple myeloma presenting with recurrent hypercalcemia in a patient with a history of primary hyperparathyroidism: report of case and review of literature. *Endocrine Practice*.

[24] Patel N, Talwar A, Donahue L, John V, Margouleff D (2005). Hyperparathyroidism accompanying multiple myeloma. *Clinical Nuclear Medicine*.

[25] Avcioglu B, Bayraktaroglu T, Kubat Uzum A (2007). A case with hypercalcemia caused by hyperparathyroidism and multiple myeloma. *Endocrine Abstracts*.

[26] Chowdhury FU, Scarsbrook AF (2008). Tc-99m sestamibi uptake mimicking parathyroid adenoma in a patient with primary hyperparathyroidism and occult multiple myeloma. *Clinical Nuclear Medicine*.

[27] Dalgleish AG, Gatenby PA (1984). Refractory hypercalcaemia: parathyroid adenoma or multiple myeloma?. *Medical Journal of Australia*.

[28] Peters KM, Rosenberger J, Gaczkowski A, Lorenz R (1989). Concomitant occurrence of hyperparathyroidism and multiple myeloma. *Internist*.

[29] Johansson H, Werner I (1975). Dysproteinemia, malignancy, and hyperparathyroidism. *Annals of Internal Medicine*.

[30] Drezner MK, Lebovitz HE (1978). Primary hyperparathyroidism in paraneoplastic hypercalcaemia. *The Lancet*.

[31] French S, Subauste J, Geraci S (2012). Calcium abnormalities in hospitalized patients. *Southern Medical Journal*.

[32] Kyle RA, Gertz MA, Witzig TE (2003). Review of 1027 patients with newly diagnosed multiple myeloma. *Mayo Clinic Proceedings*.

[33] Mundy GR, Yoneda T, Guise TA, Favus MJ (1999). Hypercalcemia in hematologic malignancies and in solid tumors associated with extensive localized bone destruction. *Primer on the Metabolic Bone Diseases and Disorders of Mineral Metabolism*.

[34] Tsujimura H, Nagamura F, Iseki T, Kanazawa S, Saisho H (1998). Significance of parathyroid hormone-related protein as a factor stimulating bone resorption and causing Hypercalcemia in myeloma. *American Journal of Hematology*.

[35] Kitazawa R, Kitazawa S, Kajimoto K (2002). Expression of parathyroid hormone-related protein (PTHrP) in multiple myeloma. *Pathology International*.

[36] John R, Oleesky D, Issa B (1997). Pseudohypercalcaemia in two patients with IgM paraproteinaemia. *Annals of Clinical Biochemistry*.

[37] Van Dijk JM, Sonnenblick M, Weissberg N, Rosin A (1986). Pseudohypercalcemia and hyperviscosity with neurological manifestations in multiple myeloma. *Israel Journal of Medical Sciences*.

[38] Clubb JS, Posen S, Neale FC (1964). Disappearance of a serum paraprotein after Parathyroidectomy. *Archives of internal medicine*.

[39] Bellou A, Blain H, Guerci A, Jeandel C (1996). Gammapathie monoclonale et hyperparathyroïdie primitive. À propos de deux observations et revue de la littérature. *Revue de Medecine Interne*.

[40] Arnulf B, Bengoufa D, Sarfati E (2002). Prevalence of monoclonal gammopathy in patients with primary hyperparathyroidism: a prospective study. *Archives of Internal Medicine*.

[41] Pirih FQ, Michalski MN, Cho SW (2010). Parathyroid hormone mediates hematopoietic cell expansion through interleukin-6. *PLoS One*.

[42] Greenfield EM, Shaw SM, Gornik SA, Banks MA (1995). Adenyl cyclase and interleukin 6 are downstream effectors of parathyroid hormone resulting in stimulation of bone resorption. *Journal of Clinical Investigation*.

[43] Luckasen JR, White JG, Kersey JH (1974). Mitogenic properties of a calcium ionophore, A23187. *Proceedings of the National Academy of Sciences of the United States of America*.

[44] Lewis EB (1963). Leukemia, multiple myeloma, and aplastic anemia in american radiologists. *Science*.

[45] Schneider AB, Gierlowski TC, Shore-Freedman E, Stovall M, Ron E, Lubin J (1995). Dose-response relationships for radiation-induced hyperparathyroidism. *Journal of Clinical Endocrinology and Metabolism*.

